# Early symptoms of bone and soft tissue sarcomas: could they be diagnosed earlier?

**DOI:** 10.1308/003588412X13171221590016

**Published:** 2012-05

**Authors:** A George, R Grimer

**Affiliations:** ^1^University of BirminghamUK; ^2^Royal Orthopaedic Hospital NHS Foundation TrustUK

**Keywords:** Sarcoma, Signs and symptoms, Referral and consultation, Delayed diagnosis

## Abstract

**INTRODUCTION:**

Delays in diagnosis are common for patients with bone and soft tissue sarcoma (STS) despite guidance produced by the National Institute for Health and Clinical Excellence. This study set out to identify early symptoms experienced by patients and reasons for delays in making a definitive diagnosis.

**METHODS:**

Retrospective interviews were carried out with 107 patients (66 with an STS and 41 with a bone sarcoma) presenting to a specialist centre. Symptoms were determined prior to definitive diagnosis and the occurrence of patient and doctor delays in reaching specialist care.

**RESULTS:**

The median patient delay was 1 month while the median doctor delay was 3.2 months from first symptoms to diagnosis for all sarcomas. Forty-nine patients with STS (74%) presented initially to their general practitioner with at least one guideline feature to prompt urgent referral. Only 2 patients (4%), however, were referred directly to a sarcoma unit, with 21 (43%) referred to secondary care for investigation. Patients with a lump increasing in size exhibited longer patient delays while doctor delay was shorter for deep lumps. Thirty-six patients with a bone sarcoma (88%) presented initially with symptoms to prompt further investigation. Nevertheless, significant delays (3.9 months) were seen in reaching specialist care. Only 4 patients (10%) were referred directly to a sarcoma unit at first presentation, with 21 (54%) referred for further investigation elsewhere.

**CONCLUSIONS:**

It is evident that awareness and referral of sarcomas remain poor. We suggest specific amendments to current guidelines and clearer referral pathways for patients. Furthermore, the need for robust education strategies is indicated, predominantly among healthcare professionals.

Bone and soft tissue sarcomas (STS) are rare and their possible diagnosis is often overlooked by healthcare professionals (HCPs). Sarcomas account for 1% of all cancer diagnoses with approximately 2,700 cases occurring annually in the UR.^1^ There is often a low clinical suspicion of malignancy and an expected benign-to-malignant ratio of 100:1 has been quoted for soft tissue lumps.^2^

Guidelines for the referral of suspected sarcomas were first introduced by the Department of Health (DH) in 2000, updated in the National Institute for Health and Clinical Excellence (NICE) guidance (2005) and reiterated by recent UR consensus guidelines:^3^^–^^5^

Urgent referral should be sought for a patient with a soft tissue mass with any one of the following features:

> size >5cm
> increasing size
> deep to the deep fascia
> painful
> recurrent after previous excision

Patients with a suspected bone sarcoma and characteristic radiographic imaging should also prompt urgent referral to a specialist centre. Suspect symptoms include: bone pain (or tenderness) of increasing intensity or that is unexplained/persistent, or an unexplained limp/pathological fracture.

Prior work has shown that for patients with a soft tissue lump, the more of these features present, the greater the risk of malignancy.^6^ DH guidelines additionally introduced a maximum two-week wait for any patient with suspected cancer to be seen by a cancer specialist.^5^ However, one study in 2007^7^ and two in 2010^8^, ^9^ have suggested little impact on clinical practice for patients with sarcomas. Poor use of two-week referral pathways has been noted. Recent work has suggested that only 15% of patients with a sarcoma are referred via this route^8^ and a large study reported a substantial median delay of 14 months in referral to their specialist centre.^9^

Delays in reaching a diagnosis of malignancy are important and lead to significantly poorer patient outcomes and prognosis.^10^ This is currently a topic of great interest with routes to diagnosis being published for the most common cancers although not for sarcomas.^11^ The most significant effect of a delay for sarcoma is that of increasing size of the lesion. This has been shown to be associated with considerably poorer patient outcomes and prognosis. The potential for uncomplicated excision with clear surgical margins decreases as tumour size increases; there is a greater risk of amputation compared with limb salvage surgery and the potential for developing metastastes.^12^, ^13^ Medical professionals are thought to confer the greatest source of delay. The reason is often a lack of clinical suspicion and frequent referral to non-specialist services contributing to delays.^13^, ^14^

Previous work looking at the symptoms of patients with sarcomas has only reported on what patients present with at the time of diagnosis and not those that might have led to diagnosis at an earlier stage.^15^ The aim of this study, therefore, was to identify what symptoms patients have both at the time of definitive diagnosis and at the time of initial presentation to an HCP, including impact on time to diagnosis. We proposed to evaluate symptoms experienced against current guidelines for referral to determine whether amendment might improve direct referral rates for patients.

## Methods

Between January and April 2011, adult patients undergoing treatment at a specialist unit with a histologically confirmed diagnosis of primary bone sarcoma or STS were invited to participate in the study. Patients were recruited from oncology clinics and surgical wards to include both newly diagnosed and review patients. A semi-structured proforma was completed for each patient to determine the initial symp toms experienced, the duration of symptoms, contact with HCPs and the subsequent investigations undertaken. This was done in retrospect covering the period from symptom onset to definitive diagnosis at a specialist centre.

Data relating to tumour characteristics were obtained from patient notes and relevant radiological imaging to include: the size at diagnosis, type of sarcoma (soft tissue/bone), histological subtype, depth and grade. Collected data were coded and analysed using SPSS® version 18 (SPSS, Chicago, IL, US). Delays in reaching specialist care were divided into patient delay (period from onset of symptoms to first presentation to a HCP) and doctor delay (period from first presentation to definitive diagnosis at a specialist centre).

**Figure 1:**
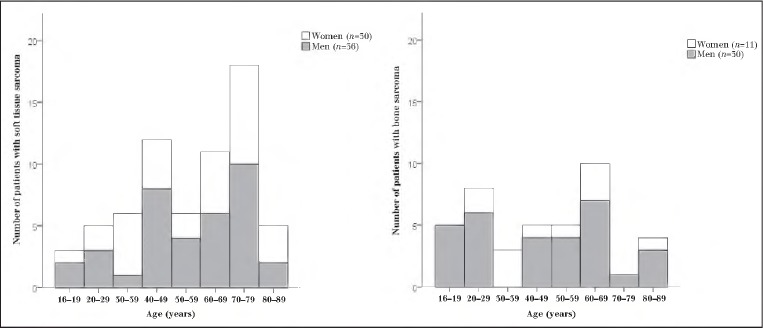
Age and sex distribution of patients for soft tissue and bone sarcoma

Of the 107 patients included in the study, 66 (62%) had an STS and 41 (58%) a bone sarcoma. There were 66 men (62%) and 41 women (58%) with a median age of 65 years for STS (range: 16–88 years) and 49 years for bone sarcoma patients (range: 17–86 years). The distribution of age and sex of participants for both STS and bone sarcomas is shown in Figure 1. The tumour characteristics are summarised in Table 1. The most common histological subtypes for STS were spindle cell and liposarcoma arising predominantly in the lower extremities. For bone sarcoma, chondrosarcoma and osteosarcoma were most common, affecting the femur, tibia and pelvis.

**Table 1:** Tumour characteristics of soft tissue and bone sarcomas

Variable	Soft tissue *(n=66)*	Bone *(n=41)*
Tumour size at diagnosis^*^		
Median	6.75cm	9.5cm
Range	1.5–30.0cm	2.5–44.0cm
Interquartile range	5.0–10.0cm	6.3–13.0cm
**Histological grade**		
High	38 (58%)	29 (71%)
Intermediate	18 (27%)	–
Low	10 (15%)	12 (29%)
**Depth**		
Superficial	28 (42%)	–
Deep	38 (58%)	–

* The overall median tumour size at diagnosis was 8cm.

A Mann-Whitney U test was used to determine significant differences in patient or doctor delay relating to each of the guideline features due to the skewed nature of the data. (A number of individuals reported excessively long delays.) Those patients with STS not reporting a lump at first presentation were excluded from analyses as guideline criteria relate to the presence of a mass. However, they were included in descriptive analyses for completeness. The significance level was set at p<0.05.

## Results

Ninety-six patients (90%) first presented to their general practitioner (GP), eight (7%) at the accident and emergency department and three to other HCPs. For STS cases, the median patient delay in reaching specialist care was 1 month (range: 0 days – 10 years) whereas the median doctor delay was 5.1 months (range: *12* days – 5 years). For bone sarcomas, the median patient delay was 1.5 months (range: 0 days – 60 years) and the median doctor delay was 5.9 months (range: 10.5 days – 4.1 years). Twenty-six patients (24%) had a 2-week wait referral at some point in their diagnostic pathway with only 8 (7%) of those being referred directly to specialist care by their GP.

### Soft tissue sarcoma

The most common reason for patients with STS to present to a HCP was a painless lump in 47 patients (71%), of whom 25 (55%) reported increasing size as a reason to prompt consultation. Each of the specified guideline features for STS were considered at the time of initial presentation. The proportion of patients presenting with each is shown in Table 2.

**Table 2:** 1 Symptoms reported and presence of guideline 1 features for soft tissue and bone sarcomas

	At first presentation to a HCP	At time of referral to specialist
**Soft tissue sarcoma**		
Size >5cm	11 (17%)	26 (39%)
Increasing size	29 (44%)	46 (70%)
Deep to the deep fascia	31 (47%)	35 (53%)
Painful lump	14 (21%)	21 (32%)
**Bone sarcoma**		
Deep (bony) pain	36 (88%)	36 (88%)
Worsening pain	17 (41%)	24 (59%)
Visible lump/swelling	20 (49%)	23 (56%)
Night pain	14 (34%)	18 (44%)
Limp/difflculty walking	12 (29%)	14 (34%)

Forty-nine patients (74%) had at least one guideline feature to prompt referral as a suspected STS at the time of initial presentation to a HCP. Twenty-two patients (45%) presented initially with only one feature, twenty (41 %) had two features, six (12%) had three features and one (2%) presented with all four features. Only 2 patients (4%) were referred directly to a specialist diagnostic clinic at their first visit while 21 (45%) were referred to secondary care. Patients underwent a median of 5 visits to HCPs (GP and specialist) prior to a diagnosis being made (range: 1–14).

Of the 59 patients with STS who presented with a lump at first presentation, only H (17%) reported this to be larger than 5cm. Twenty-six patients (44%) had a lump >4cm at first presentation while 55 (59%) had a lump >5cm. Six patients (9%) did not present initially with a lump. AH of these presented with pain and were later diagnosed with tumours deep to the fascia. Three of these patients reported no presence of a lump at the time of definitive diagnosis.

Delays in diagnosis relating to each of the specified guideline features were assessed using univariate analysis. A lump of increasing size was the only significant guideline feature associated with longer patient delay (median: 45.5 vs 14 days, p=0.002). For doctor delay, the only significant factor was depth. Those patients with lumps deep to the fascia experienced shorter doctor delays compared with those with superficial lumps (median: 70 vs 152.9 days, p=0.058). All comparisons for both patient and doctor delays for each of the specified guideline criteria are displayed in Table 3.

**Table 3:** Median delays experienced by patients with soft tissue sarcomas relating to the presence of guideline features

	Median patient delay	*p*-value	Median doctor delay	*p*-value
**Increasing size**				
Yes (n=29)	45.5 days		102.8 days	
No(n=30)	14.0 days	**0.002**	85.8 days	0.982
**Painful**				
Yes (n=13)	30.4 days		74.9 days	
No(n=46)	30.4 days	0.912	100.4 days	0.37
**>5cm**				
Yes (n=ll)	14.0 days		70.0 days	
No (n=48)	30.4 days	0.119	104.1 days	**0.09**
**Depth**				
Deep (n=31)	30.4 days		70.0 days	
Superficial (n=28)	30.4 days	0.665	132.9 days	**0.038**

### Bone sarcoma

Symptoms experienced by patients with bone sarcomas both at first presentation to an HCP and at time of referral to a specialist unit are outlined in Table 2. The most common reason to prompt initial consultation was deep (bony) pain in 29 patients (71%), with 8 of those (21%) also reporting worsening pain as a reason to prompt consultation.

Thirty-six patients (88%) with bone sarcomas presented with features to prompt further investigation and/or referral at first presentation to an HCP. Of those, 21 patients (54%) were referred for further investigation. However, only four (10%) were referred to a specialist unit directly. Patients underwent a median of 5 visits to HCPs prior to a diagnosis being made (range: 1–9).

An additional four patients reported difficulty moving a limb in the upper extremity at first presentation to an HCP, with a further three patients at the time of referral to a specialist centre. Only three patients (7%) reported a history of a pathological fracture prior to referral to our centre. None of the factors for bone sarcoma were associated with a significant difference in either patient or doctor delays.

### All patients

Twenty-three patients (21%) reported a previous history of trauma related to the sarcoma location although no significant difference in patient delay (median: 50.4 days for both trauma and no trauma, p=0.71) or doctor delay (median: 96 days for trauma vs 100.4 days for no trauma, p=0.78) was found. Thirty-four patients (52%) were referred to our unit following inappropriate biopsy and/or excision. However, there was no significant delay in this group (median: H5.8 vs 87.5 days, p=0.552).

## Discussion

This study has set out to explore delays in the presentation and referral of patients with sarcoma and whether current guidelines are adequate for appropriate referral to specialist services. We found HCPs to confer the greatest source of delay (5.1 months for STS and 5.9 months for bone sarcoma). Johnson *et al,^14^* however, reported a longer delay of 5.7 months for STS and Ashwood *et al^16^* reported a delay of 7.5 months for bone sarcoma and STS. This is likely to reflect differences in data collection or study populations. Ninety per cent of patients first presented to their GP, many with features already set out in current guidelines for early referral. When patients were referred, it was to the ‘correct’ place in only 7% of cases. The remainder were either reassured or sent to the local hospital for evaluation.

### Soft tissue sarcoma

Of the 66 patients eventually found to have an STS, 71% presented initially with one or more guideline features to prompt referral to a specialist centre. A lump of increasing size actually deterred patients from seeking healthcare (longer patient delays), although 55% reported this as a reason to consult suggesting a possible trigger. Conversely, only a deep mass tended to prompt HCPs to refer patients quickly. Interestingly, absolute size (greater or less than 5cm) did not significantly impact either patient or doctor delays.

It has been suggested that any lump larger than a golf ball (42mm) should have a diagnosis.^12^, ^17^ Had this criterion been adopted, 44% of those presenting with a lump would have been referred directly for investigation and diagnosis on the basis of size alone. We therefore recommend any lump >4cm should, at the very least, be investigated properly to reach a definitive diagnosis. All other lumps should be actively monitored and referral should be sought following any subsequent increase in size.

### Bone sarcoma

The most common presenting feature for patients with bone sarcoma was deep pain (88%), reported as worsening in 41%. There are of course many reasons for musculoskeletal pain. Nevertheless, pain that is worsening, in particular, should be investigated. Additionally, unexplained difficulty using either the upper or lower limb should prompt further concern. Current guideline criteria only allude to the presence of a limp.

Night pain is not a guideline feature currently despite being present in 54% of patients at initial consultation and in 44% by the time of referral to a specialist centre. Widhe and Widhe reported only 20% experiencing night pain.^18^ However, this is likely to reflect the relatively selective nature of their population (limited to osteosarcoma or Ewing sarcoma and those aged <50 years). Furthermore, a palpable lump/swelling was present in 49% of our patients at first presentation while Widhe and Widhe reported this in over a third of patients.^18^ The inclusion of night pain and a lump or swelling may therefore be valuable amendments to current guidelines in reducing delays for bone sarcoma. A small proportion (7%) also reported a pathological fracture, consistent with previous work suggesting between 5% and 10%.^19^

Although approximately half of those patients with bone sarcoma were referred for further assessment at initial presentation, there were still significant delays (5.9 months) until they reached specialist care. This suggests that although some HCPs may refer patients quickly, they may be referring them to the wrong places. Only four patients (10%) were referred directly to our specialist unit. Clearer guidelines to aid appropriate investigation and diagnosis are therefore indicated.

### All patients

Tumour size is cited consistently as one of the most significant prognostic factors for sarcoma and that most amenable to change by HCPs. For every centimetre increase at the time of definitive diagnosis, there is an estimated 5–5% reduction in overall survival for STS.^20^ The average tumour size at diagnosis in our study was slightly lower than in previous work but this may be an artefact due to the majority of studies reporting mean rather than median size. The mean size for our cohort (8.2cm for STS and 10.7cm for bone sarcoma) is comparable with other series, with articles reporting minimal change in size at diagnosis over the past 20 years.^17^, ^20^

A third of patients underwent inappropriate biopsy and/or inadequate excision. These patients are likely to have residual disease or contaminated resection margins requiring further wide local excision at a specialist centre.^21^^–^^24^ Appropriate investigation and management carried out by a dedicated oncological multidisciplinary team has been shown to improve both patient survival and quality of life substantially.^25^^–^^28^ Evidence from a Swedish study has also demonstrated the value of targeted education strategies for sarcoma among students and HCPs in reducing improper investigations or management.^22^ The use of ultrasonography to screen suspicious soft tissue lumps has also been suggested. However, there have been mixed results as to its usefulness as a diagnostic modality for musculoskeletal malignancy.^29^, ^30^

This is the first study of its kind to evaluate early symptoms of both soft tissue and bone sarcomas. The main limitation was its retrospective nature, making it subject to significant recall bias. Furthermore, only patients presenting to a specialist centre were included. However, due to the small proportion treated elsewhere, the impact of this is likely to be minimal. Additionally, no paediatric patients (<16 years) were included although a significant peak of bone sarcomas arises in early adolescence.^1^ This is reflected in the study population; there was a greater proportion of patients with chondrosarcoma rather than the more common osteosarcoma or Ewing sarcoma. Further work to validate our results in a larger series and in the paediatric population is therefore indicated.

## Conclusions

Despite the introduction of national guidelines more than a decade ago, prompt referral of patients with a possible sarcoma to specialist care remains poor and often results in delays. Although patients do contribute to delays, this is difficult to reduce as widespread public awareness will likely lead to undue public concern and an unnecessary diagnostic workload for HCPs. We therefore urge greater awareness of potentially malignant lesions and strategies to improve awareness among HCPs. This is particularly so for inexperienced or non-specialist surgeons who are most likely to carry out a biopsy and/or excision of presumed benign pathology.^31^

We believe future research should review the additional criteria we have identified to see if these can lead to earlier diagnosis. In particular, we recommend all lumps >4cm should be investigated to obtain a diagnosis, and anyone with bone pain and reduced function of the limb or with night pain should be investigated for a bone sarcoma. We have incorporated these features into possible new guidelines:

Urgent referral to a sarcoma diagnostic clinic should be sought for patients with a soft tissue mass with any one of the following features:

> size >4 cm
> increasing size
> deep to the deep fascia
> painful
> recurrent after previous excision

Patients with bone sarcomas may have the following symptoms:

> bone pain (typically of increasing intensity, not related to activity and that may wake them at night)
> bone tenderness or swelling
> difficulty using the limb or an unexplained limp
> unexplained pathological fracture

Any of these should prompt referral for plain film radiography of the affected part or referral for investigation.

## Acknowledgements

The authors would like to thank Dr Lesley Roberts for support in the production of this manuscript and Mr Roger Holder for input on statistical methodology. Furthermore, the authors would also like to thank members of the multidisciplinary team involved in patient care who made this study possible.
